# Editorials

**Published:** 1911-01

**Authors:** 


### EDITORIALS

The Business Office of the *Journal-Record* is 1 1-2 to 5 1-2 South Broad Street.
The Editorial Office is 1014-15 Century Building.
Address all Business Communications to *Journal-Record of Medicine*, 1 1-2 to 5 1-2 South Broad Street.
Make remittances payable *to The Journal-Record of Medicine.*
On matters pertaining to the Editorial and Original Communications, address Edgar G. Ballenger, M. D., Atlanta, Ga.
Reprints of Original Articles will be furnished at cost price. Requests for the same should always be made in THE MANUSCRIPT.
We will present, postpaid, on request, to each contributor of an original article, twenty (20) marked copies of *The Journal-Record of Medicine* containing such article.

### DIRECTIONS FOR USING "606" OR SALVARSAN.

Salvarsan or "606" has now been placed upon the market within the reach of any physician who may care to use it. While this remedy is a wonderfully potent one and will undoubtedly prove a great blessing to humanity, it has its limitations and contra-indications and requires a rather elaborate and careful technique to give it properly and a word of warning and full directions concerning its administration seem appropriate at this time. The writer has been rather surprised at the large number of patients who applied for treatment with it who showed no signs of syphilis, nor did they present reasonably clear histories of ever having had the disease. As the usefulness of salvarsan is largely limited to luetic infection, it is urgenty advised that a correct diagnosis be made and that the Wasserman reaction be determined before this treatment is given. Furthermore, it is even more important to examine the patient to see the condition of the optic nerve and to eliminate affections of the heart, kidney, liver, brain, spinal cord, lungs, etc. Nor should the remedy be administered and the patients then dismissed; the treatment has been in use too short a time to know about the ultimate results and every patient should be carefully watched for two or three years, even after all lesions have been cured. If the second dose is necessary it should be given in three or four weeks after the first treatment.

The suggestions and directions which follow are those recommended by Ehrlich:

"*Chemical-Physical Properties*: Salvarsan is a light-yellowish powder, which contains about 34 per cent. arsenic and dissolves in water forming strongly acid solutions. On account of the acid reaction these solutions are unsuitable for injections, and must be neutralized before use in accordance with the directions given below.

*Indications*: Salvarsan is indicated in the treatment of primary, secondary and tertiary syphilis and accompanying symptoms, as well as for preventive measures. An important field for the employment of the remedy is afforded by cases of lues maligna and obstinate affections of the mucous membrane. Particularly favorable results have been obtained in those cases which were refractory to iodine and mercury.

Salvarsan has also been employed with remarkable success against the syphilis of pregnant and nursing women, as well as in hereditary lues. According to present experience the medicament can only be used with a prospect of success in incipient tabes, early paralysis and epilepsy, of syphilitic origin, if the treatment is commenced immediately after the very earliest symptoms appear. Recurrent fever and indeed all spirillar diseases, malaria and marsh fever, are diseases in which treatment with Salvarsan is likewise indicated. Even in severe cases of pemphigus, lichen ruber planus, framboesia and psoriasis, and in disorders of the blood and nervous system, where arsenical medication appears indicated, the drug may also be experimentally tried.

*Contra-Indications*: The medicament is contra-indicated in serious disturbances of the circulatory system, advanced degeneration of the central nerve system, foetid bronchitis, as well as in cachexia unless a direct consequence of syphilis. It is also contra-indicated in patients who exhibit a pronounced idiosyncrasy against arsenic. On the other hand, according to Lesser, Michaelis, and Spiethoff, diabetes, nephritis and tuberculosis are not contra-indications.

Although hitherto no case of visual disturbance after employment of Salvarsan has been reported, the strictest precautions should be observed in treatment of existing opthalmic disorders, even if of luetic origin.

*Dosage*: According to Michaelis the average dose is 1 centigramme (0,01 gramme) per kilo bodyweight (1 1-14 grain per lb), the nearest whole number above the total calculated dose being selected if the general condition of the patient is good.

From the clinical records it appears that if smaller doses than 0,5 gramme ( 7 1-2 grains) are injected, the remedial action may be incomplete and lead to relapses. Experience has shown that for adult males of strong constitution, according to the nature of the case, doses of 0,6—0,7—0,8 and 1,0 gramme (9, 10 1-2, 12 and 15 grains) may be employed. For women smaller doses usually suffice, from 0,45 to 0,5 gramme (6 3-4 to 7 1-2 grains) upwards. For weakly or very enfeebled patients doses of 3,0 to 0,4 gramme ( 5 to 6 grains) and for children 0,2 to 0,3 gramme (3 to 5 grains) are indicated.

For suckling infants doses of 0,02—0,05—0,1 gramme (1-7, 3-4 and 1 1-2 grains) have been employed with the best results. In the earliest stages of incipient tabes and in disorders of the nerves and blood, doses of 0,3 to 0,4 gramme (5 to 6 grains) suffice.

*Mode of Employment*: Salvarsan may be injected subcutaneously, intramuscularly or intravenously. It should be mentioned that the previous administration of mercurials does not stand in the way of employment of Salvarsan, and that the mercurial treatment can again be followed after conclusion of the Salvarsan treatment. Various observations point to the conclusion that Salvarsan and mercury mutually support each other's action.

In subcutaneous injection the injection is made between the shoulder-blades at the side of the vertebral column and in a downward direction, or beneath the skin of the breast. If an interscapular injection is to be made, the patient should be directed to stretch the arm backwards, in order that a loose fold of skin can easily be raised. On the breast a fold of skin below the nipple should be selected in men, and below the mammary gland in women. Care should always be observed that the subcutaneous injection is made *lege artis,* and not partially intracutaneous, otherwise a lasting infiltration may result. Subcutaneous injection should not be employed for youthful patients, those whose skin sits very close and is not easily stretched, nor in cases where the skin is badly nourished, nor for very young children. In such cases intramuscular injection is preferable.

Intramuscular injection is made in the external upper parts of the gluteal region. The injection should be deep, but made very slowly so as to avoid tearing and hemorrhage of the muscles.

The injected fluid after subcutaneous or intramuscular injection should be distributed by careful massage over as large an area as possible, and a moist dressing placed over the locality.

It is advisable for the patient to remain in bed for 2 or 3 days after injection, under the supervision of a reliable nurse.

*Intravenous Injections*: According to the most recent experience the intravenous injection of Salvarsan is distinctly superior to all other methods of injection, and it is therefore thoroughly to be recommended. With proper technique no unpleasant local reactions set in at the point of injection.

The average dose for intravenous is:

for women . . . 0,3 gramme Salvarsan.
for men . . . . 0,4 gramme Salvarsan.

It would be impracticable to inject larger quantities than 0,5 gramme Salvarsan intravenously. The intravenous injections of an equal dose should be repeated after 3 or 4 weeks. No anaphylactic symptoms have been observed even after repeated injections.

Salvarsan is at present supplied in ampulae containing 0,6 gramme. This quantity is dissolved in the following manner:

30 to 40 c. c. sterile physiological saline solution (0,9%) which is prepared from chemically pure sodium chloride and distilled water, are poured into a graduated sterile glass stoppered measure with a narrow neck and 300 c. c. capacity, containing about 50 sterile glass beads. Then 0,6 gramme Salvarsan is added. By means of vigorous shaking the substance is dissolved. To this solution, according to above table, 23 minims of 15% sodium hydrate solution are added. This will cause a precipitate to form, which is again dissolved by vigorous shaking. Then the clear yellow solution ensuing is increased to 300 c. c. by adding sterile physiological saline solution. Should the solution not be quite clear, another 1 or 2 minims of the sodium hydrate solution are added.

Each 50 c. c. of this solution now contain 0,1 gramme Salvarsan, therefore 150 c. c. 0,3 gramme 200 c. c. 0,4 gramme and 250 c. c. 0,5 gramme.

The intravenous injections of this solution is carried out either with the usual syringes or by means of a burette tapering at the lower end, having a capacity of 250 c. c. and graduated to 50 c. c. An India rubber tube is attached to the burette, the flow of the solution is controlled by a clamp and a canula is inserted at the end of the tube. By releasing the clamp a few drops of the solution are allowed to escape in order to remove the air from the tube and canula, after which the latter is inserted into the vein. The flow of the solution into the vein is regulated by raising or lowering the burette.

In the absence of a graduated measure with glass beads the solution of Salvarsan may also be prepared in a small sterile mortar as follows:

The contents of one ampoulla (0,6 gramme Salvarsan) are emptied into a small, sterile mortar. Then with the aid of a sterile drop pipette 23 minims of a 15% sodium hydrate solution are dropped direct on the powder. By mixing the powder and sodium hydrate with a small sterile pestle or a thick rounded glass rod, a clear yellow solution is at once formed, which is then also diluted to 300 c. c. with physiological saline solution.

The above instructions for the intravenous application of Salvarsan can naturally possess no claim for general application. The intensity of the treatment must necessarily be regulated by the individual state of the disease of each patient, and by the degree of infection. From the literature hitherto published, however, one can safely say that primary chancres and especially the secondary early stages of syphilis require a specially intensive treatment. In the cases of patients suffering from affections of the central nerve system and cardiac troubles, even in the event of such patients proving suitable for treatment, caution is certainly indicated and this should be expressed by employing smaller doses (0,2 to 0,3 gramme). If the first injection was stood well the injection of this small dose may be repeated if necessary in a few days. Whether intravenous injection should also receive preference over other methods of application in these cases, cannot yet be definitely decreed; at all events the intravenous injection should certainly not be administered in the case of patients with severe cardiac troubles.

*Preparation of Neutral Suspensions for Subcutaneous and Intramuscular Injection*: The greatest care must be exercised in the proper preparation of Salvarsan suspension, as thereupon depend the painlessness of the injection, the therapeutical efficacy and the absence of by-effects. The following utensils are required for the preparation of the neutral suspensions:

1 small porcelain dish.

1 thick glass rod with rounded ends,

1 bottle containing caustic soda solution and a pipette.

(Rp. Purified Sodium Hydroxide 1, 5 gramme

Distilled Water . . . . . 8,5 cubic centimetres).

1 drop-bottle containing diluted hydrochloric acid

(Rp. Acid hydrochlor. dilut. officinal. (10,58% Brit. Pharm.) ad. vitr. patent. 10 c. c.)

Red and blue litmus paper.

If, for instance, it is desired to make an injection of Salvarsan 0,6 gramme (9 grains), the procedure is as follows:

The quantity of 0,6 gramme is weighed out into the porcelain dish and triturated carefully with 0,54 gramme=0,456 c c. (specific gravity 1,17), corresponding to about 9 or 10 drops of the 15% caustic soda solution. Then there is added with constant stirring, at first drop by drop, the desired volume (about 5 to 10 c. c.) sterilized water. The fine suspension which is formed is tested with litmus paper exactissime for neutral reaction. According to the litmus reaction a drop of caustic soda or of hydrochloric acid is added.

The suspension, the preparation of which is quite easy and only occupies a few minutes, must be injected immediately after its preparation by means of a record syringe with a very thick platinum needle. The strictest aseptic precautions must be taken in the preparation of the suspension. The locality of the injection must previously be disinfected with iodine-benzine or iodine tincture.

According to Kromayer a suspension of Salvarsan in paraffin is also suitable for subcutaneous injection, this may be prepared, for example, by triturating the contents of an ampul of 0,6 gramme, *lege artis,* with paraffin liquid, sterilisat. and filling up to 6 c. c.

With sensitive patients the locality of the injection may be completely anaesthetised by a prior injection of 2 c. c. of a 1 per cent. Novocain solution. Hydropathic measures, such as moist dressings, hip-baths, etc., or even the application of warmth can be successfully adopted to relieve or prevent any after-pains or painful infiltrations.

*Preparation of Alkaline Solutions for Intravenous Injection*: The "suspension" described above must not be employed for intravenous injections, but a completely clear solution, absolutely free from suspended matter, must be used.

With intravenous application of Salvarsan, the arsenic has been completely eliminated from the organism after about 3 or 4 days, whilst the elimination of arsenic after subcutaneous or intramuscular injection occupies a considerably longer time. For this reason a number of clinicians have tried to combine the "intensive" and "permanent' action of both methods of injection in this way, that at first an intravenous injection of 0,4 to 0,5 gramme Salvarsan is made, followed after 2 or 3 days with an intramucular or subcutaneous injection of 0,4 to 0,5 gramme.

*Warning*: Salvarsan is supplied to us only in ampuls which have been evacuated and then filled with an indifferent gas, so as to protect the medicament from oxidation processes.

We urgently warn against the employment of suspensions or solutions which have not been prepared immediately before use. The colour of Salvarsan must be light yellow. Discoloured preparations with a greyish or brownish appearance, must not be used.

Neither use contents of ampuls damaged in transport, nor the remainder of the drug left in ampuls previously opened, must be used, as it involves danger to the patient."

The writer has treated sixteen patients by the intravenous and subcutaneous methods as above described without any harmful or disagreeable effects, and with very prompt healing of visible symptoms, but it is too early to judge as to the ultimate cure.

E. G. BALLENGER, M. D.

---

### THE AMERICAN SOCIETY OF MEDICAL SOCIOLOGY.

Recognizing the intimate relationship between disease and the social-economic system under which we live, recognizing that many diseases are caused directly by our social and economic conditions, recognizing that the efficiency or inefficiency of our treatment often depends upon the economic condition of the patient, recognizing that there are many problems deeply and vitally affecting the welfare of mankind which are left practically untouched by any existing medical society, The American Society of Medical Sociology has been organized for the purpose of studying radically all questions of a socio-medical nature, and invites your co-operation.

Some of the questions which are under investigation by the members at the present time are:

The need of a Federal Department of Health.

Tuberculosis as an economic disease.

Is there any demonstrable relationship between the strain of our modern life and the increase of insanity?

Is cancer on the increase, and if so, what are the probable etiologic factors?

What are the best, i. e., the most human and most effective methods of dealing with prostitution?

The best methods of preventing venereal infection?

Is complete sexual abstinence (a) likely to impair the general health? (b) likely to result in impotence?

The relative influence of heredity and environment on the physical, mental and moral characteristics of the offspring.

The question of marriage and divorce.

Is the regulation of conception morally justifiable, and if so, what are the best methods?

Abortion in its medical and ethical aspects.

Alcohol (a) as a beverage, (b) as a medicine. Its physiologic, medicinal, social and economic effects.

Infant mortality. Its principal causes and prevention.

Occupational or trade diseases.

Food adulterations and their influence on health.

The causes of quackery, Christian science and other cults, and the influence of the irregular cults of medicine on public health.

The results of these investigations will be disseminated by means of meetings, lectures, reports, pamphlets, etc.

If you wish to become an earnest student of socio-medical questions and wish to join the Society, you will please fill out the enclosed blank and send it to

